# Supplementary material for: Direct observation of the Migdal effect induced by neutron bombardment
Source: Nature. 2026 Jan 14;649(8097):580–3. doi: 10.1038/s41586-025-09918-8 (PMC12804083; doi:10.1038/s41586-025-09918-8)
Supplement: Supplementary file 1 — This file contains Supplementary Notes 1–4 and additional references. [file 41586_2025_9918_MOESM1_ESM.pdf]

nature portfolio

https://doi.org/10.1038/s41586-025-09918-8

## Supplementary information

# Direct observation of the Migdal effect induced by neutron bombardment

In the format provided by the authors and unedited

## Supplementary Information

## Note 1. The total probability of Migdal effect:

Under the soft limit (the electron transfer momentum is much lower than the transfer momentum of the nucleus), the neutron Migdal effect scattering cross-section can be factorized into two parts:

$$\frac{d\sigma_{\text{Migdal}}}{dE_r dE_e} = \sum_i \frac{d\sigma_s^i}{dE_r} \times \sum_{nl} \frac{dp(nl \to E_e)}{dE_e} \quad (1),$$

where $d\sigma_s^i/dE_r$ is the neutron-nucleus cross-section which includes elastic cross-section and inelastic cross-section and $dp(nl \to E_e)/dE_e$ is the transition probability. For the four elements C, H, He and O, the neutron-nucleus scattering is still dominated by elastic scattering at the neutron incident energy of 2.5 MeV, so we can only use the elastic cross-section safely. Since the experimentally detected quantity is the Migdal electron probability, which is:

$$\frac{dp_{\text{Migdal}}}{dE_r dE_e} = \frac{1}{\sigma_{tot}} \frac{d\sigma_{elastic}}{dE_r} \times \sum_{nl} \frac{dp(nl \to E_e)}{dE_e} \quad (2),$$

where the $\sigma_{tot}$ is the total neutron-nucleus scattering cross-section.

After integrating the nuclear recoil energy $E_r$, the differential probability can be expressed as:

$$\frac{dp_{Migdal}}{dE_e} = \frac{1}{\sigma_{tot}} \int_{E_r^{th}}^{E_r^{Max}} \frac{d\sigma_{elastic}}{dE_r} \times \sum_{nl} \frac{dp(nl \to E_e)}{dE_e} dE_r \quad (3),$$

where $E_r^{th}$ is the experimentally allowed nuclear recoil energy threshold and $E_r^{Max}$ is the maximum nuclear recoil energy.

Finally, the Migdal probability can be expressed as:

$$P_{Migdal}^{tot} = \int_{E_e^{th-Min}}^{E_e^{th-Max}} \frac{dp_{\text{Migdal}}}{dE_e} dE_e \quad (4),$$

where $E_e^{th-Max(Min)}$ is the experimentally allowed electron recoil energy maximal (minimal) threshold which is 10 keV (5 keV) in our experiment.

We utilize the scheme described in the Theory section of the Methods to calculate the Migdal cross-section. ENDF/B-VIII.0 library is used as the result of nuclear cross sections. For the transition probability of H, He and C, we consider the semi-inclusive transition probability from Ref.[1] which is applicable to high-energy neutron incidence. What needs to be declared here is that the transition probability for H agrees with that for He in the case of high-energy incident neutrons, so we use semi-inclusive transition probability of He to replace H. For O, since the calculation of the semi-inclusive ionization probability does not include the O element, we adopt the electron boost method[2], that is, using the neutron-electron scattering form factor to replace the Migdal ionization probability. The result can be expressed as:

$$\frac{dp(nl\to E_e)}{dE_e} = \frac{dp(nl\to E_e)}{d\ln E_e}\left|\frac{d\ln E_e}{dE_e}\right| \Rightarrow \frac{1}{E_e}\frac{\pi}{2}\left|f_{nl}^{ion}\left(p_e, \frac{m_e}{m_N}q\right)\right|^2 \quad (5),$$

where $p_e$ and q are the electron and nuclear momentum. $f_{nl}^{ion}$ is neutron-electron scattering form factor which is the result of integrating the radial wave function of the $n$ and $l$ bound state orbitals, the continuous wave function and the spherical Bessel function of the first kind. We have proved that the result of this method in high-energy neutron scattering is consistent with those of semi-inclusive ionization probability. Therefore, this method can be used with confidence. The corresponding differential probabilities of Migdal electron emission are shown in Extended Data Fig. 1 **a**,**b**,**c**,**d**.

## Note 2. Neutron and Environmental Gamma Spectra Measurement:

The neutron flux and spectrum of the D-D neutron generator are measured and monitored using a Φ2″ ×2″ EJ309 Liquid Scintillator (LS) detector, capable of discriminating fast neutrons from gamma rays[3]. Calibration of the detector involves multiple radioactive gamma sources to determine its energy linearity and resolution curves. The neutron flux and spectrum are obtained by applying an unfolding method to the measured neutron energy deposition spectrum.

### EJ309 detector calibration

The EJ309 detector is calibrated using four radioactive gamma sources: $^{241}$Am, $^{133}$Ba, $^{137}$Cs, and $^{60}$Co. By fitting either the Compton edge (at energies of 200 keV for $^{133}$Ba, 477 keV for $^{137}$Cs, and 963 keV and 1118 keV for $^{60}$Co) or the full energy deposition peak (at 60 keV for $^{241}$Am) created by these mono-energetic gammas in the LS detector, the scaling factor between true energy deposition and ADC integral value, along with the resolution at certain energies, can be determined. The fitting model comprises three components. Firstly, a Monte Carlo simulation using GEANT4 is performed to simulate the true energy deposition spectrum in the detector due to gammas of specific energies. Its shape is extracted using the RooKeysPdf toolkit in CERN ROOT (Etruth). Secondly, a Gaussian function (Gausres) with $\mu = 0$ and $\sigma$ as a free-floating parameter is convolved with the true energy deposition PDF to represent the energy resolution of the detector. Finally, a scaling factor (scale) is introduced to characterize the scale difference between the true energy deposition and ADC integral (PeakIntegral), as illustrated in equation 6. A fitting example is shown in Extended Data Fig. 3 **a**.

$$E_{\mathrm{exp}} = \mathrm{scale}\times(E_{\mathrm{truth}}\otimes Gaus_{\mathrm{res}}) \quad (6).$$

With all the fitted parameters obtained at different energy points, the energy linearity and resolution curves can be determined. The relationship between ADC integral (PeakIntegral) and true energy is described by a linear function, as shown in equation 7, where $k$ is the proportionality constant and d represents the non-linearity effect at low energies for the LS detector[4]. Extended Data Fig. 3 **b** depicts the fitting result.

$$E = k \times PeakIntegral + d \qquad (7).$$

The energy resolution of an LS detector can be parameterized as shown in equation 8. Here, $\Delta E$ represents the σ of the Gaussian representing resolution at energy $E$. α denotes the intrinsic energy resolution of the detector, which arises from the nonuniformity of the scintillator and differences in light collection efficiency for light generated at different positions. $\beta$ is associated with the statistical uncertainties of the photons generated in the LS and is typically around 1. $\gamma$ represents the noise from electronic systems and the dark counts of the photomultiplier. Extended Data Fig. 3 **c** illustrates the fitted energy resolution curve.

$$\frac{\Delta E}{E} = \sqrt{\alpha^2 + \frac{\beta^2}{E} + \frac{\gamma^2}{E^2}} \qquad (8).$$

**Pulse shape discrimination between neutron and gamma**

The EJ309 LS detector can distinguish fast neutrons from gammas based on their waveform characteristics. Typically, neutron signals exhibit longer tails in the waveform compared to gamma signals. A Pulse Shape Discrimination (PSD) factor is employed to separate neutrons from gammas, defined by equation 9, where $A_{\mathrm{tail}}$ represents the waveform integral of the signal from 50 ns to 210 ns from the trigger point, and $A_{\mathrm{total}}$ is the waveform integral from the trigger point to 210 ns after. Extended Data Fig. 3 **d** illustrates the selection of neutron signals based on 2D distributions of PSD and energy.

$$PSD = \frac{A_{tail}}{A_{total}} \qquad (9).$$

**Neutron flux and spectrum unfolding**

By applying the neutron selection method outlined in the PSD section, we can obtain the neutron energy deposition spectrum. This spectrum, along with the true energy spectrum and the number of incident neutrons, can be determined using iterative unfolding algorithms. A dedicated unfolding toolkit has been developed based on the GRAVEL and MLEM algorithms[5]. Statistical errors and the correlation matrix of the unfolded spectrum are obtained using the bootstrap method. The response matrix of the EJ309 detector is acquired through GEANT4 simulation, utilizing the neutron response function measured in Ref.[6] for an EJ309 detector of the same size (see Extended Data Fig. 3 **e**, **f**).

To validate the unfolding method, the detector is positioned at approximately 87° with respect to the D−D beam. The neutron energy deposition spectrum is depicted in Extended Data Fig. 3 **g**. Several initial spectrum guesses are tested to evaluate systematic uncertainties associated with the choice of initial spectrum. The Gauss guess assumes a Gaussian distribution with μ = 2.5 MeV and σ = 0.5 MeV for the initial spectrum, representing a good estimate of the expected neutron spectrum, given that neutrons generated in the D-D neutron generator have a mean energy of approximately 2.5 MeV. Conversely, the Plain guess assumes a uniform distribution initially, representing no prior knowledge before unfolding. Additionally, GRAVEL and MLEM

algorithms are tested to assess uncertainties related to the choice of algorithm.

The unfolding process inherently accounts for detector efficiency. Integrating the unfolded spectrum allows for the determination of the initial incident neutron count on the LS detector, with its statistical uncertainty accurately assessed by equation 10, where the σ is the vector of bin errors and the ***Cor*** is the correlation matrix (Extended Data Fig. 3 **i**). Furthermore, by considering the effective surface area of the EJ309 detector, its distance from the neutron source, and the relative neutron yield curve at different angles as documented in Ref.[7], the neutron flux at a specific position can be calculated.

$$\sigma_{\text{total}} = \vec{\sigma} \cdot Cor \cdot \vec{\sigma} \quad (10).$$

**Note 3. Background:**

**Bremsstrahlung processes**

After the neutron scattering, the generated recoil nucleus will interact with the nearby atoms and molecules, resulting in acceleration process and produce x-ray, which then undergoes photoelectric effect. If the photoelectron is generated near the nucleus recoil vertex with proper energy, it can introduce a Migdal-like fork topology.

In general, the spectrum of these four bremsstrahlung processes exhibits a layered structure within the continuum X-ray spectrum. Specifically, the QFEB, SEB, AB, and NB processes predominate in distinct energy regions of the X-ray spectrum, as follows[8],[9]:

$$\omega < T_r = m_e \frac{E_p}{m_p},$$

$$T_r < \omega < T_m = 4m_e \frac{E_p}{m_p},$$

$$T_m < \omega < \omega_{AB} = \frac{2m_e \alpha Z_T v_p}{\left( z_p Z_T \frac{m_e}{m_p} \left( 1 - \frac{m_p Z_T}{m_T z_p} \right) \right)^{1/4}},$$

$$\omega_{AB} < \omega < \omega_{NB} = \frac{m_T}{m_T + m_p} E_p \quad (11),$$

where $m_e$, $m_p$, and $m_T$ are the masses of electron, projectile (NR), and target atom, respectively. $E_p$ and $v_p$ are the kinetic energy and velocity of the projectile. $z_p$ and $Z_T$ are the charge number of the projectile and the atomic number of the target atom, respectively. Once the photon energy $\omega$ exceeds the corresponding maximum energy threshold, the intensity of bremsstrahlung rapidly decreases, such as the SEB within the X-ray spectrum region above $T_m$, where $T_m$ represents the maximum energy transferable

from the projectile to a free electron.

In this study, we rely on Ref.[10]-[12] to obtain the theoretical differential cross-sections for the four bremsstrahlung processes induced by protons or light ions with few MeV kinetic energies. These results are presented in Extended Data Fig. 6 **b**, **c**, **d**, **e**, with specific focus on the outcomes induced by 2.5 MeV protons. The recoil energy of $^{1}$H (approximately 2.5 MeV) is significantly greater than that of $^{12}$C, where the endpoint energy of the recoil spectrum of the latter is about 0.71 MeV. According to the equation 11, we can ascertain that the $T_m$ for recoiling hydrogen and carbon nuclei is approximately less than 5 keV. Therefore, we consider that the major bremsstrahlung background of Migdal effect in X-ray spectrum region 5-10 keV are the AB and NB induced by the recoiling hydrogen and carbon. In general, the intensity of AB and NB is small and can be considered negligible in the region of interest of X-rays when induced by lower velocity proton beams. However, the X-rays from AB and NB are proportional to the square and quartic of ion charge number for different NR at the same velocity. Therefore, the contribution of carbon ions needs to be considered in this work.

To estimate the expected number of electrons induced by these four bremsstrahlung processes, we extract the expected number of X-ray emission and X-ray emission energy spectrum of each type of nucleus from the differential cross section of bremsstrahlung. The spectrum is then put into GEANT4 to simulate the photoelectric process. The 200 μm vertex cut for nuclear recoil and photoelectron is applied. Since the photoelectric process distributes X-ray's energy into photoelectron and nucleus, both of which are detectable, the 5-10 keV energy cut is applied to X-ray instead of photoelectron.

**Random track coincidences**

The recoil nuclei produced by neutrons may coincidentally overlap with Compton electrons or photoelectrons, thereby forming pseudo-Migdal events and contributing to the background. In the background analysis process, GEANT4 is employed for simulation, with the detector materials and structure. This portion of the background is categorized into three sources: a) Single-Neutron-Induced Coincidence; b) Multi-Neutron-Induced Coincidence; c) Neutron-Gamma Mixed Coincidence (see Extended Data Fig. 6 **f**, **g**, **h**).

**Single-Neutron-Induced Coincidence**

After a neutron collides with a gas atom within the sensitive volume of the detector and generates a recoil nucleus, the neutron can still generate gamma through inelastic scattering or (n, γ) reactions, leading to the production of Compton electrons or photoelectrons within the sensitive volume. These Compton electrons or photoelectrons may coincidentally overlap with the recoil nucleus, forming pseudo-Migdal events.

Using GEANT4, 50 billion neutrons are simulated to be incident normally on the detector. Among these, recoil nuclei generated within the sensitive volume of the detector are selected, taking into account the quenching effect and requiring the recoil nucleus energy to exceed 50 keV. A total of 3,860,962 recoil nuclei are found.

Additionally, Compton electrons and photoelectrons with entire tracks confined within the sensitive volume of the detector are selected, with energies ranging between 5 and 10 keV, resulting in 208 electrons. The probability of randomly generated electron tracks and recoil nucleus tracks within a single frame being identified as Migdal events by the selection algorithm is determined to be 0.0029. Consequently, the background contribution from Single-Neutron-Induced Coincidence is calculated to be 0.1276±0.0089(stat.), normalized to $8.17\times 10^5$ recoil nucleus events.

**Multi-Neutron-Induced Coincidence**

Neutron interactions with the shielding material or the detector can generate gammas, which may subsequently induce the production of Compton electrons or photoelectrons within the sensitive volume of the detector. Notably, even in cases where the neutrons responsible for generating these electrons do not produce recoil nuclei, there exists a probability that these electrons may spatially and temporally coincide with a recoil nucleus, sharing the same event frame and vertex.

A simulation is conducted using GEANT4, employing a 4π neutron source that emits 1 trillion neutrons isotropically toward the shielding material. Within the sensitive volume of the detector, recoil nuclei are selected with an energy threshold exceeding 50 keV, yielding a total of 126,797 recoil nuclei. Concurrently, Compton electrons and photoelectrons with their entire trajectories confined to the sensitive volume and energies ranging from 5 to 10 keV are identified, resulting in a total of 279 electrons. The relative ratio of the production probability of Compton electrons and photoelectrons to that of recoil nuclei is quantified as 0.00220. Considering the probability of an electron track and a recoil nucleus track coinciding within the same frame (0.00363) and the probability of successful coincidence between two randomly selected tracks within the same frame (0.0029), the final background contribution, normalized to $8.17\times 10^5$ recoil nucleus events, is calculated to be 0.0189±0.0011(stat.).

**Neutron-Gamma Mixed Coincidence**

Gammas from the environment have a probability of generating Compton electrons or photoelectrons within the detector. These electrons may coincidentally appear in the same frame as a recoil nucleus and be misidentified as Migdal events, thereby contributing to the background.

Since the liquid scintillator is positioned 1.8 m from the neutron source while the GMPD detector is located 0.4 m from the neutron source, the gamma flux ratio between the GMPD detector and the liquid scintillator is 16.7. Based on the gamma flux and energy spectrum detected by the liquid scintillator, simulations indicate that during the experimental period, the number of Compton electrons and photoelectrons generated by environmental gamma in the detector within the energy range of 5 to 10 keV is 701.6 and 161.1, respectively. The probability that an electron track and a recoil nucleus track are located in the same frame is 0.00363, and the probability of a successful coincidence between any two randomly selected tracks within the same frame is 0.0029. Consequently, the background contribution from this component is determined to be 0.0091±0.0004(stat.), normalized to $8.17\times10^5$ nuclear recoils.

The total background obtained from the aforementioned simulations and analyses is 0.156±0.009(stat.), showing excellent agreement with the data-driven background estimation of 0.180±0.022(stat.)±0.042(sys.).

**Gas radioactivity:**

For the analysis of trace radioactive background, the sources are systematically categorized into two distinct groups: the first group comprises isotopes of hydrogen, helium, carbon, and oxygen, while the second group encompasses all other trace radioactive nuclides excluding H, He, C, and O. This classification facilitates a more structured and precise evaluation of the radioactive contributions.

Among the isotopes of H, He, C, and O, $^{3}H$ and $^{14}C$ exhibit relatively high abundances, with estimated values of $10^{-18}$ and $10^{-12}$, respectively, both accompanied by an uncertainty of 100%. The half-life of $^{3}H$ is 12.32 years, while that of $^{14}C$ is 5730 years. Based on:

$$A = {ln(2)N_A}\Big/{T_{1/2}M} \quad (12),$$

where $A$ is specific activity, $N_A$ is Avogadro's constant, $T_{1/2}$ is the half-life, and $M$ is the molar mass, the specific activities of $^{3}H$ and $^{14}C$ are calculated to be 0.00106± 0.00106 Bq/g H and 0.192±0.192 Bq/g C, respectively. In a gas mixture of 0.8 atm consisting of 40% He and 60% DME with a sensitive detection volume of 6×6×14 $mm^3$, the activities of $^{3}H$ and $^{14}C$ are $(6.38\pm6.38)\times10^{-8}$ Bq and $(4.58\pm4.58)\times10^{-5}$ Bq. According to the $\beta$ decay spectrum, the probabilities of emitting electrons with 5-10 keV are 0.359 for $^{3}H$ and 0.0565 for $^{14}C$. Consequently, the rates of $^{3}H$ and $^{14}C$ generating 5-10 keV electrons per second within the sensitive detection volume are $(2.29\pm2.29)\times10^{-8}$ and $(2.59\pm2.59)\times10^{-6}$, respectively.

The trace radioactive background that produces 5–10 keV electrons, except for H, He, C, and O in the environment, is almost entirely contributed by $^{222}Rn$. To achieve a more accurate estimation of the trace radioactive background, a novel approach is employed to directly evaluate the frequency of 5-10 keV electron production from trace radioactive nuclides except H, He, C, and O within the detector. The approach involves operating the detector in a dark chamber for six days to collect data. Given that the trace radioactive background is predominantly due to $^{222}Rn$, and that the decay chain of $^{222}Rn$ produces MeV-scale alpha particles, the number of ion tracks in the collected data can be utilized to estimate the activity of $^{222}Rn$ in the working gas. This activity estimation is then applied to evaluate the frequency of 5-10 keV electron production.

Based on the measurement data from the detector's 6-day operation in a dark chamber, a total of 60 ion tracks within the sensitive volume are identified. It is noteworthy that numerous factors can contribute to the formation of these ion tracks, such as atmospheric neutrons. However, for the most conservative background estimation, all selected ion tracks are assumed to originate from alpha particles emitted during the decay of $^{222}Rn$. For $^{222}Rn$ decay chain, secondary nuclides with half-lives

shorter than one year are considered to fully decay, ensuring a more robust and conservative background estimation. Consequently, a single $^{222}$Rn decay event results in the emission of 3 alpha particles and 2 electrons, ultimately concluding in $^{210}$Pb with a half-life of 22.3 years. Based on this, the activity of $^{222}$Rn in the working gas is determined to be 20±3 decays over six days. Using $\beta$ decay spectra from $^{214}$Pb, $^{214}$Bi, and $^{210}$Tl), the probability of generating 5-10 keV electrons from $^{222}$Rn decay is found to be 0.0188. Therefore, the number of 5-10 keV electrons produced per second due to trace radioactivity within the sensitive volume is calculated to be $(7.25\pm0.94)\times10^{-7}$.

In summary, the number of 5-10 keV electrons generated in the sensitive detection volume per second by trace radioactivity is determined to be $(3.34\pm2.71)\times10^{-6}$.

Through GEANT4 simulations of ion tracks in the working gas and electron tracks generated by trace radioactivity in the energy range of 5-10 keV, pseudo-Migdal events are simulated. The proportion and energy of ions are determined based on the elemental composition of the working gas and the neutron energy, while the electron energy is sampled from the beta decay spectra of $^{3}$H, $^{14}$C, $^{214}$Pb, $^{214}$Bi, $^{210}$Tl. A total of 100,000 pseudo-Migdal events are simulated, and after applying the Migdal event selection algorithm, 11,439 events are selected. This results in a selection efficiency for pseudo-Migdal events induced by trace radioactivity of 0.114±0.016. In conclusion, the trace radioactivity background is estimated to be 0.00106±0.00087 events, normalized to the total number of nuclear recoil events of $8.17\times10^{5}$.

## Note 4. YOLO:

### Motivation and Applications of YOLO:

In the initial batch of experimental data, candidate events of the Migdal effect are manually identified. However, this approach suffers from extremely low efficiency, making it impractical to analyze a big dataset rapidly, while the uncertainties introduced by human judgment remain unquantifiable. To address these limitations, YOLO (You Only Look Once), a deep learning-based image recognition tool, is employed, which significantly enhances efficiency and enables systematic error estimation.

The implementation of YOLO is inspired by the work of the MIGDAL Collaboration[13]. However, unlike their approach, YOLO is utilized solely for the identification of images containing at least one NR and one ER track.

### Image Preprocessing

Label Studio is used for data annotation. The detector's readout employs Topmetal with a pixel resolution of 72×72. However, 72×72 images are too small for efficient labeling in Label Studio, so they are proportionally upscaled to 288×288 pixels. To achieve optimal visualization, a logarithmic color bar is applied to the pixel ADC values, ranging from 10 to 2600, with a gradient from blue to red.

### Classification and Labeling

The Migdal effect exhibits a unique topological signature characterized by ER and NR sharing a common vertex. In Label Studio, only two classes are defined: ER and NR, as no other classes are required. The reasons are as follows:

- Cosmic rays, sparks, and hot pixels. These are filtered out by separate algorithms, eliminating the need for manual labeling.

- Protons and alpha particles. First, distinguishing them from NRs is unnecessary because their track morphologies closely resemble NRs, and the experimental gas contains hydrogen, which can produce protons. Second, protons, alpha particles, and NRs cannot be completely distinguished due to charge adsorption effects, which cause observable variations in dE/dx along tracks. This implies that NRs are not purely bare nuclei, and their charge states remain uncertain. Without experimentally calibrated training data of NRs, relying solely on simulated bare-nucleus samples introduces significant model bias.

- Ghost tracks. During preprocessing, adjacent frames with signals are algorithmmically merged into complete tracks. Pixel-wise calibration of the attenuation pattern is performed, and attenuation corrections between adjacent frames are incorporated, as detailed in the Digitization section (PixelControl and TopmetalControl classes) of the Star-XP. This approach inherently eliminates ghost tracks.

**Dataset**

The training set contains approximately 6,000 real data points and 2,400 simulated data points. Half of the real data points are $^{55}$Fe photoelectrons, and the other half are D-D source recoil NRs; half of the simulated data points are 4-10 keV ERs, and the other half are simulated NRs. The validation set includes about 2,800 real data points and 600 simulated data points, with the proportion and distribution of data types consistent with those of the training set.

**Training and Validation**

Initially, different YOLOv8 model architectures (e.g., YOLOv8n and YOLOv8m) are evaluated to balance accuracy and training efficiency. After comprehensive analysis, YOLOv8m is selected as the optimal architecture. For dataset augmentation, 90°, 180°, and 270° rotations, as well as horizontal and vertical flipping, are applied, while some simulated data is incorporated for transfer learning.

After training, the model's performance is validated using a validation dataset. Extended Data Fig. 8 **a**, **b** show the confusion matrix and F1 curve of the model. Extended Data Fig. 8 **c** shows how the loss functions gradually converge with the increase of training epochs.

**Efficiency, Error and Results**

When validating the model's accuracy using the validation dataset, very few images contain both ER and NR tracks. Thus, these results cannot directly represent the efficiency of selecting Migdal effect candidates. To evaluate YOLO's efficiency in

selecting Migdal candidates, Star-XP is used to simulate $1.5\times10^5$ events of the Migdal effect (one ER and one NR per event) within the 5-10 keV ER energy range, based on the theoretical Migdal effect cross section. After applying YOLOv8m, 29.3% of events are retained where the model identifies at least one ER and one NR. Subsequently, the Migdal event selection algorithm further filters these candidates, achieving an additional 49.0% efficiency. The final reported efficiency of 14.4% is the product of these two efficiencies.

Regarding YOLO's systematic error, the discrepancy between YOLOv8m and YOLOv8n models trained on identical datasets and parameter configurations is quantified. After applying YOLOv8n, 25.6% of events are retained where the model identifies both ER and NR tracks. Subsequent filtering via the Migdal event selection algorithm achieves an additional 51.8% efficiency. Compared to YOLOv8m, this results in a 1.9% systematic error in the final efficiency. Extended Data Fig. 5 display the YOLO-predicted bounding boxes for the 6 selected Migdal effect events.

## References


[1] Cox, P., Dolan, M. J., McCabe, C., & Quiney, H. M. Precise predictions and new insights for atomic ionization from the Migdal effect. *Phys. Rev. D* **107(3)**, 035032 (2023).

[2] Essig, R., Pradler, J., Sholapurkar, M., & Yu, T. T. Relation between the Migdal effect and dark matter-electron scattering in isolated atoms and semiconductors. *Phys. Rev. Lett.* **124(2)**, 021801 (2020).

[3] Eljen Technology. Neutron/gamma PSD: EJ-301, EJ-309. (2024). https://eljentechnology.com/products/liquid-scintillators/ej-301-ej-309

[4] Dietze, G., & Klein, H. Gamma-calibration of NE 213 scintillation counters. *Nucl. Instr. and Meth.* **193(3)**, 549–556 (1982).

[5] Chen, Y., et al. Unfolding the fast neutron spectra of a BC501A liquid scintillation detector using GRAVEL method. *Sci. China Phys. Mech. Astron.* **57**, 1885-1890 (2014).

[6] Bai, H., et al. Calibration of an EJ309 liquid scintillator using an ambe neutron source. *Nucl. Instr. and Meth. A* **863**, 47-54 (2017).

[7] Zhang, S. Y., et al. Measurement of neutron source characterization of the compact D–D neutron generator with unfolding algorithm. *Eur. Phys. J. A* **59(5)**, 101 (2023).

[8] Ishii, K. High energy limit of atomic bremsstrahlung. *Nucl. Instr. and Meth. B* **99(1)**, 163-165 (1995).

[9] Ishii, K. Continuous X-rays produced in light-ion-atom collisions. *Radiat. Phys. Chem.* **75(10)**, 1135-1163 (2006).

[10] Chu, T. C., Ishii, K., Yamadera, A., Sebata, M., & Morita, S. Quasifree electron bremsstrahlung induced by 20-MeV-proton impact. Phys. Rev. A 24(4), 1720 (1981).

[11] Ishii, K., & Morita, S. Theoretical estimation of PIXE detection limits. *Nucl. Instr. and Meth. B* **34(2)**, 209-216 (1988).

[12] Ishii, K., et al. Atomic bremsstrahlung of Al, Ag and Au targets bombarded with 1.5 MeV protons. X-Ray Spectrom. 37(2), 121-124 (2008).

[13] Schueler, J., et al. Transforming a rare event search into a not-so-rare event search in real-time with deep learning-based object detection. Preprint at https://arxiv.org/abs/2406.07538 (2024).
